# Reef Odor: A Wake Up Call for Navigation in Reef Fish Larvae

**DOI:** 10.1371/journal.pone.0072808

**Published:** 2013-08-28

**Authors:** Claire B. Paris, Jelle Atema, Jean-Olivier Irisson, Michael Kingsford, Gabriele Gerlach, Cedric M. Guigand

**Affiliations:** 1 Applied Marine Physics, Rosenstiel School of Marine and Atmospheric Science, University of Miami, Miami, Florida, United States of America; 2 Marine Program, Boston University, Boston, Massachusetts, United States of America; 3 Observatoire Océanologique, Station Zoologique, Laboratoire Oceanographique de Villefranche-sur-Mer, Villefranche-sur-Mer, France; 4 Australian Research Council Centre of Excellence for Coral Reef Studies and the School of Marine and Tropical Biology, James Cook University, Townsville, Australia; 5 Oldenburg University, Oldenburg, Germany; 6 Marine Biology and Fisheries, Rosenstiel School of Marine and Atmospheric Science, University of Miami, Miami, Florida, United States of America; The Australian National University, Australia

## Abstract

The behavior of reef fish larvae, equipped with a complex toolbox of sensory apparatus, has become a central issue in understanding their transport in the ocean. In this study pelagic reef fish larvae were monitored using an unmanned open-ocean tracking device, the drifting *in-situ* chamber (DISC), deployed sequentially in oceanic waters and in reef-born odor plumes propagating offshore with the ebb flow. A total of 83 larvae of two taxonomic groups of the families Pomacentridae and Apogonidae were observed in the two water masses around One Tree Island, southern Great Barrier Reef. The study provides the first *in-situ* evidence that pelagic reef fish larvae discriminate reef odor and respond by changing their swimming speed and direction. It concludes that reef fish larvae smell the presence of coral reefs from several kilometers offshore and this odor is a primary component of their navigational system and activates other directional sensory cues. The two families expressed differences in their response that could be adapted to maintain a position close to the reef. In particular, damselfish larvae embedded in the odor plume detected the location of the reef crest and swam westward and parallel to shore on both sides of the island. This study underlines the critical importance of *in situ* Lagrangian observations to provide unique information on larval fish behavioral decisions. From an ecological perspective the central role of olfactory signals in marine population connectivity raises concerns about the effects of pollution and acidification of oceans, which can alter chemical cues and olfactory responses.

## Introduction

Despite the critical role of larval behavior in scaling population connectivity revealed theoretically by coupled biological and physical models [1], [2], our ability to understand their movement in response to the conditions they experience in the pelagic realm remains limited to indirect observations of their vertical migration, using invasive techniques of plankton surveys, e.g. [3], [4].

Behavior of minute fish larvae equipped with a complex toolbox of sensory apparatus [5] has thus become a central issue in understanding their transport in the marine environment. There is increasing evidence that the pelagic larval stages are receptive to cues that might guide them toward suitable settlement habitat [6]. This sensory capability is critical to surviving the early pelagic stages and recruiting to the benthic population. Nearly every aspect of larval fish behavior examined thus far has produced surprising evidence of the sophistication and range of larval behavioral abilities [7]. Yet, the few studies tackling the sensory abilities of fish larvae test the behavior of late stage larvae by scuba [8], or are carried out in laboratories [9], [10] and inferred from numerical models [11], [12]. Almost nothing is known about the orientation of larvae far offshore and their response to cues *in situ*.

Coastal habitats possess unique signatures [13], [14]. Several suggested that reef smells could be utilized primarily for a homing function, as demersal species may be imprinted at birth and thus more attracted to the smells of their home reef than other reefs [15], [16]. The quest of salmonid fishes for their native river at the time of reproduction exemplifies the ability of aquatic animals to use olfaction for homing [17]. Coral reef fish larvae must also find a specific benthic habitat to survive as juveniles, at the end of their pelagic phase, and navigate actively in the ocean using various signals. Sound is probably an important directional cue [18] but only within an acoustic perception range for fish larvae of a few hundred meters [19] with a detection limit of reef ambient noise just over 1 kilometer [20]. Odor is exquisitely suited to determine the identity of its source and has been related to gradient maps in the air. But olfactory signals in the water are more complex since molecular diffusion is *ca*. 10,000 slower in water than in air (translated in hundred-fold distance disadvantage) [21]. Instead, chemical signals are dispersed in the ocean by currents and associated eddy diffusivity. Consequently fish larvae are not subjected to continuous odor gradient but to turbulent and high intermittency in odor signal [22]. Thus, while odor gradients are vectors, they may not be useful for orientation in the sea, and other senses need to come into play. We know that in choice tests, coral reef fish larvae prefer lagoon odor to oceanic odor [15], and even that the chemical signature of individual coral reefs is highly specific and can be transported many kilometers offshore in turbulent flows [16], [22]. Larvae dispersed offshore should thus smell the reef before they can hear it.

To understand the chemo-sensory guidance of their homing behavior, we need to test whether and how larvae use these capabilities directly in the ocean. Studying the behavior of mm-sized fish larvae remains a major research frontier as they are too small for remote tracking [23]. Direct diver observation has yielded interesting results but provided limited insight as to the stimuli used [24]. Therefore, to overcome these problems, and with the goal to determine the behavioral responses of fish larvae to reef-born chemical cues in their natural settings, we used a novel Lagrangian observational framework [25]. Despite their small size, we could monitor the movement of pelagic reef fish larvae using an unmanned open-ocean tracking device. Here, we present the first evidence that reef fish larvae under field conditions use reef odor as a component of their navigation.

## Materials and Methods

The *One Tree Island Research Station* from the University of Sydney approved the permits for this study. Permit numbers or approval ID for this study are the following: Great Barrier Reef Marine Park Authority (GBRMPA): GIO 33.239.1 and Fisheries Permit DPI 10 32 56. Larvae were caught at night in light traps and crest nets and tested the day following.

**Figure 1:**
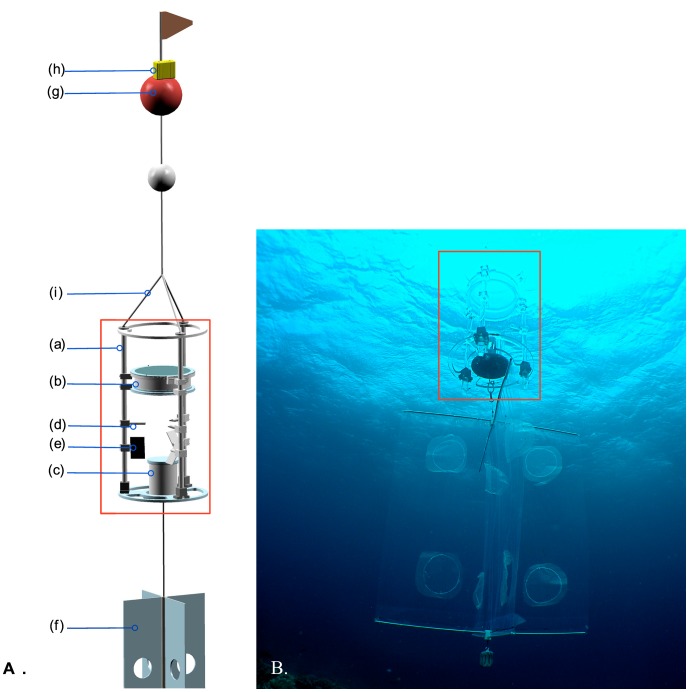
The Drifting In Situ Chamber (DISC). A) schematic view of the Lagrangian observational system. The hardware includes a main underwater unit [red rectangle] composed of a cylindrical frame (H 1.2 m× ∅ 0.63 m) made of clear acrylic bars (a) holding a behavioral mesh chamber (∅ 0.38 m, mesh-size ∼1 mm) (b), a pressure enclosure (c) housing an electronic compass and the imaging system composed of a camera with high capacity memory card, a time lapse, and a large battery, allowing for up to 8 hours of continuous recording at 1 HD frame per second. Other instruments include an analog compass (d) and a mini-CTD (e) that senses the ambient conductivity, temperature, and depth. The underwater unit is locked into the current by a drogue (f) and connected to a surface float (g) and Global Positioning System (GPS) (h) by a 3 mm-diameter nylon line attached with three stainless steel bridles (i) to the top ring of the underwater unit; the length of the line is adjusted the target deployment depth. B) *In situ* view of the DISC deployed off One Tree Island (OTI), Great Barrier Reef. The immersed underwater unit is symmetrical and becomes transparent, minimizing visual disturbances to the tested larva. Graphic courtesy of Bellamare LCC [drogue and surface float not to scale]; photo credit M. Kingsford.

**Figure 2:**
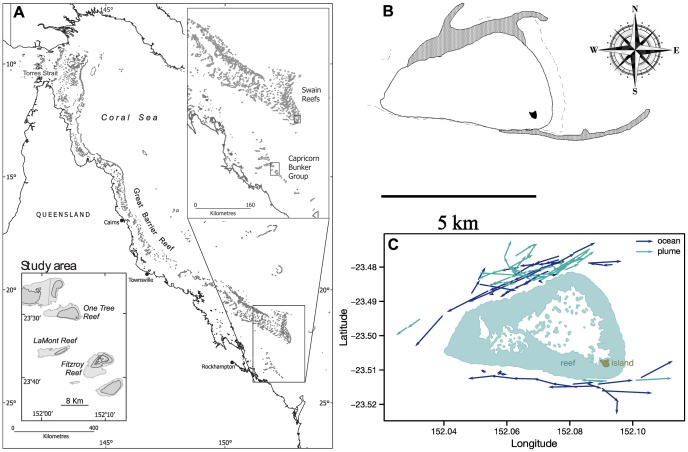
Sampling Site and DISC deployments. A) One Tree Island (24°30′ S, 152° E, teal square) belongs to the Capricorn Bunker Reef at the southern end of the Great Barrier Reef at the edge of the Coral Sea; B) double odor-plumes (gray shade) observed during Northwest-North winds and ebb flow; B) twenty-minute trajectories of the DISC deployed north and south of One Tree island (OTI) in ocean (dark blue) and plume water (green). The ebb flow (green trajectories) is mainly towards the north-northeast on the northern side of OTI and towards the east on the southern side. A total of 83 DISC trajectories are shown for February 8–22, 2009.

Pre-settlement stage larvae from two common reef fish families (cardinalfish, Apogonidae, and damselfish, Pomacentridae) were placed individually in a Drifting In Situ Chamber (DISC, Fig. 1) equipped with a circular arena, open to ambient water and transparent to turbulence [24]. A camera looking up at the chamber monitored how individual fish larvae responded behaviorally to different water masses around One Tree Reef, Australia (Fig. 2). We chose One Tree Reef for this study because it produces conspicuous ebb-tide plumes, which can be mapped with GPS while driving a boat along visible frontal margins [26] (Figure 2B). These turbidity plumes carry reef odor into the ocean and make it simple and credible to deploy the test chamber in either reef odor or ocean odor. In addition, One Tree is the outermost reef of the Capricorn-Bunker reef group and borders the open ocean (Fig. 2A) making the distinction between reef and ocean water stand out.

Previous work at OTI showed that pomacentrid and apogonid larvae in flume tests can sense the difference between ocean and lagoon water preferring the lagoon odor [15]. For this study, we deployed the DISC in two natural environmental conditions: 1) in the green plume water mass carrying lagoon water offshore at ebb tide, and 2) in the blue ocean water mass outside the odor plume (Fig. 2). Deployments were done over 9 days of sampling during which we tested 42 Apogonidae belonging to four species (Gymnapogon sp., Cheilodipterus quinquelineatus, Apogon cyanosoma, A. doederleini) and 41 Pomacentridae belonging to five species (Abudefduf sp., Chromis atripectoralis, Dascyllus sp., Pomacentrus coelestis, P. moluccensis). No fish larva was tested repeatedly (Table 1).

**Table 1:** Summary of the data collection and analysis.

	Apogonidae		Pomacentridae	
	ocean	plume	ocean	plume
Initial sample size	25	17	20	21
Average swimming speed (cm s^−1^)	**1.4**	**1.7**	**1.5**	**1.0**
Student’s t-test	**t = −1.78, p = 0.082**		**t = 2.12, p = 0.041**	
Significant directionality (Sample size for orientation tests)	20	11	18	20
Population bearing (degree East, from North)	241°	309°	303°	**268°**
Rayleigh test	r = 0.22 *p* = 0.37	r = 0.28 *p* = 0.43	r = 0.22 *p* = 0.41	**r = 0.48 ** ***p*** ** = 0.008**
Orientation w/r reef crest (degree right from the reef crest)	146°	33°	133°	**98°**
Rayleigh test	r = 0.17 *p* = 0.57	r = 0.09 *p* = 0.93	r = 0.22 *p* = 0.41	**r = 0.51 ** ***p*** ** = 0.005**
Orientation w/r current (degree right from the bearing of the current)	317°	175°	192°	40°
Rayleigh test	r = 0.32 *p* = 0.13	r = 0.18 *p* = 0.71	r = 0.33 *p* = 0.14	r = 0.31 *p* = 0.14
Orientation w/r wind (° right, from the bearing of the wind)	182°	105°	210°	118°
Rayleigh test	r = 0.33 *p* = 0.11	r = 0.15 *p* = 0.80	r = 0.21 *p* = 0.54	r = 0.39 *p* = 0.11

Of the 83 larvae observed, 83% showed significant directionality (significant first order Rayleigh test). At the population level, swimming speeds were different between plume and ocean for both families (10% and 5% confidence levels for Apogonidae and Pomancentridae, respectively). Orientation was significant for Pomacentridae larvae in plume water only and was Westward (268°), while swimming alongshore (nearly perpendicular to the direction of the reef atoll, at 98°).

### 1. Lagrangian Observation Procedure

The observational system used in this study is an improved version of the Orientation With No-apparent Frame Of Reference (OWNFOR) experimental prototype designed to collect information about navigational cues in marine larvae [25]. A major modification of the instrumentation included the placement of the behavioral chamber above the structure with the imaging system looking up to capture skylight cues. The resulting Drifting In Situ Chamber (DISC, Fig. 1) is a hollow cylinder structure (H 1.2 m, ∅ 0.63 m) made of acrylic rods, rigged with a circular behavioral arena near the top (∅ 0.38 m) and an underwater imaging system with digital camera, time-lapse, and compass at the bottom (Fig. 1Ac). The transparency and density of acrylic makes the device almost neutrally buoyant and inconspicuous underwater. The chamber is made of translucent molded mesh (*ca*. 1 mm) and is therefore open to larger scale turbulent flow and chemical cues, and transparent to sound. The DISC is linked to a surface float bearing a Global Positioning System (GIS) and to a drogue underneath that keeps it locked in the current, drifting with the water mass in which it is embedded. A subsurface float on elastic line enhances the decoupling of the underwater unit from surface waves. Its operating principle is similar to the prototype described in [25]. For this study, the camera recorded still images of the position of the larva every second, which were geo-referenced cardinally by an electronic compass. The data were analyzed with custom statistical software [27].

### 2. Experimental Protocol

**Figure 3:**
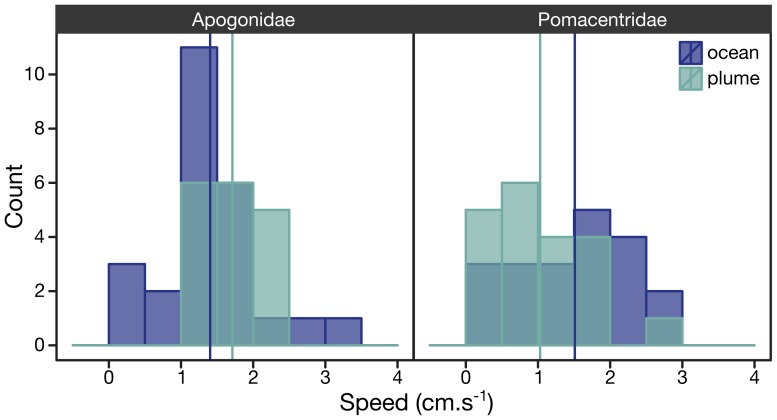
Larval Activity. Histograms of *in situ* swimming speed frequencies computed for individual larvae of the Apogonidae (left panel) and Pomacentridae (right panel) families are compared between ocean (blue) and plume (green) water masses. The data are normally distributed and the mean indicated by the vertical solid lines were significantly different for both families (t-test, Table 1); the distributions of speeds in ocean and plume water were significantly different (Kolmogorov-Smirnov, p<10-4) for the Pomacentridae.

**Figure 4:**
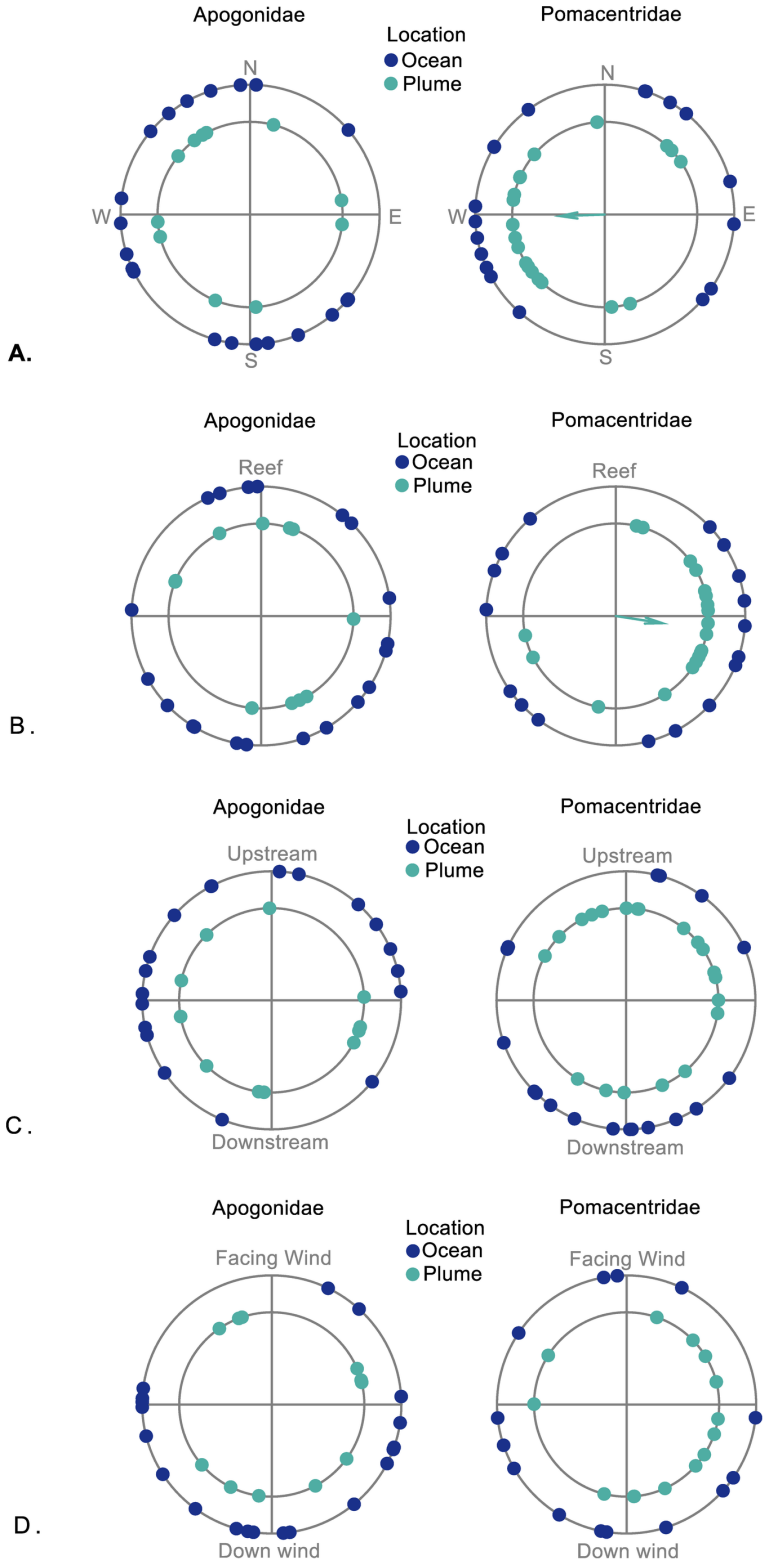
Larval Orientation. Orientation for Apogonidae (left panels) and Pomacentridae (right panels) larvae compared between ocean (blue) and plume (green). Larval mean individual bearings were computed relative to (A) a cardinal frame of reference (B) the reef crest coastline (C) the direction of the current and (D) the direction of the wind. Apogonid orientation was never significant when tested in a cardinal reference or relative to the reef crest. Pomacentrids were significantly oriented only in plume water, cardinally towards the West (p = 0.008) and alongshore when viewed relative to the reef crest (p<0.005). Each dot on the graphs represents an average individual bearing. Orientation at the population level is significant when all the larvae tested have statistically similar bearings; this is shown with an arrow pointed in the mean orientation direction of individual-level bearings, the length is proportional to the r-value.

Observations were carried out on both sides of OTI reef in February 2009 (Fig. 2). The following procedure was repeated for all larvae tested. Firstly, the DISC’s underwater unit was immersed upward next to a small boat, a larva was placed into the behavioral arena by opening the top circular, mesh plate of the chamber. Secondly, the DISC was gently turned on its side under water to hook the drogue. Lastly, the DISC was slowly released by reeling out a line until the surface float-GPS system was on the water surface. The DISC was deployed for 20 min periods (allowing for 5 min acclimation +15 min observation), in either reef water (odor plume) or ocean water. Once the DISC was deployed with the arena sitting at 3 m from the surface, the boat was taken upwind and the motor was shut down. After 20 minutes the DISC was retrieved, the larva was released and replaced by a new test animal [25]. Afterwards, image analysis [27] generated mean swimming speeds (Fig. 3) and mean bearing for each individual (Fig. 4). The 20-min drift of the DISC recorded with the GPS gave a measure of the current direction and strength during each deployment (Fig. 2B).

### 3. Movement Analysis and Statistics

In enclosed circular arenas, orientation movement is typically indicated by the position of the animal corresponding to its bearing [28]. Further, the activity of the animal can be obtained form the analysis of the trajectory [29]. It is important to note that the magnitude of swimming speeds recorded in enclosed arenas are typically lower than that of free swimming fish larvae (e.g., *30*) and are only indicative of relative levels of larval activity between treatments. Here we used larval positions recorded every second to compute and statistically quantify their swimming speed [27]. To remove autocorrelation of positions, data were subsampled at 10 s intervals with random starting time. We then used partial bootstrapping and repeat this process 1000 times for each larva [25]. The percentage of sub-sampling is chosen as the largest percentage for which independent data are obtained.

#### Swimming speed

Data slower than sample rate (≤0.4 cm s^−1^) were discarded [27]. We computed individual larval mean speed (Fig. 3) and tested for significant difference between the two test conditions (i.e., reef vs. ocean water) using t-test to compare mean speeds and the Kolmogorov-Smirnov’s two samples test to compare speed distributions (Table 1). We considered the two fish families (Pomacantridae and Apogonidae) separately.

#### Orientation behavior

The bearings were first used to assert the directionality of each individual, i.e., the concentration of the fish larva positions relative to the center of the arena around an average heading, using the Rayleigh test of uniformity (first order analysis [31] (Fig. 4). The rotation of the DISC measured by the compass allowed us to convert the larval positions in the chamber’s frame of reference (before correction by the compass’ readings) to their positions in the cardinal reference (after correction by the compass’ readings) [27]. Then we tested for orientation at the population level (Table 1). Larvae that were not keeping a significant bearing were discarded. Mean bearings of directional larvae were used as data in a second order Rayleigh test [31], to assert the orientation of the population of larvae for each treatment (i.e., reef and ocean water) towards a common heading direction. We considered the two fish families (Pomacantridae and Apogonidae) separately.

Sample size for circular statistics was at least 5 [32]. All analyses were performed in R [33], using version 0.3–8 of the “circular” package [34].

## Results

Of 83 DISC deployments, 35 were in reef water (‘plume’), 45 in oceanic water (‘ocean’), and 3 were in the front between the plume and the ocean and were included in the ‘plume’ treatment. Most larvae (69 out of 83, 83%) showed significant directionality (i.e., individuals kept a bearing, significant first order Rayleigh test) in reef or ocean water (Table 1). Those that were not directional (14 larvae) moved in a random fashion and were included in the swimming speed analysis but removed form the orientation analysis.

**Figure 5:**
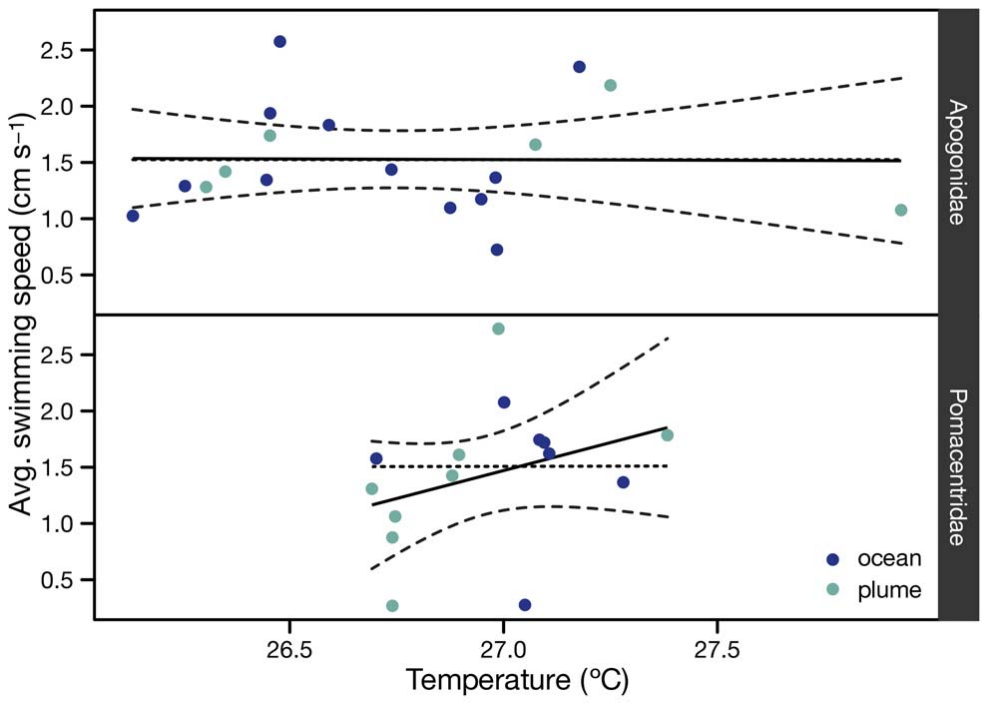
Influence of temperature. Relationship between temperature and larval swimming speed. Points are temperature averages per deployment. Solid lines are least square regression lines simple linear regression. Grey areas are 95% confidence intervals. The fact that the zero slope dashed lines stay within the confidence intervals shows visually that the regressions are not significant (*p* = 0.97 for Apogonidae, *p* = 0.22 for Pomacentridae).

At the population level, swimming speeds were different between plume and ocean, albeit only at the 10% confidence level for Apogonidae (Table 1). Indeed, apogonid larvae swam faster in the odor plume (Fig. 3A) but without a preferred overall bearing (i.e., no orientation at the population level) in either plume or ocean (Fig. 4A). In addition, orientation direction of apogonids was never significant (Rayleigh r always <0.4, *p* always >0.1, Table 1) when tested relative to the direction towards the reef outer margin, the direction of the current or of the wind, in both treatments (Fig. 4B–D). We considered that increased activity of apogonid larvae in the reef water could have been related to a physiological response to warmer temperature (overall temperature in plume 27.5°C vs. ocean 26.8°C; Wilcoxon, W = 451, *p* = 0.04). However, a regression analysis did not show a significant relationship between swimming speed and temperature, both averaged per deployment (Fig. 5). The absence of relationship was observed for both families (Fig. 5, Apogonidae (*p* = 0.97), Pomacentridae (*p* = 0.22). The activational effect of reef water on the apogonids larvae was thus unrelated to temperature.

In contrast, pomacentrid larvae swam significantly (*p* = 0.041) slower in the odor plume (Fig. 3B, Table 1) presumably carrying reef odor [16]. In addition, in the plume, they oriented significantly westward (268°) (Fig. 4A, Table 1) and alongshore on either sides of the island (Fig. 4B, Table 1). We considered that alongshore orientation might be related to the direction of the currents in the odor plume (Fig. 2B). However, the individual bearings were never related to the direction of the current as measured by the drift of the DISC (Rayleigh r always <0.4, *p* always >0.1, Fig. 4C). Interestingly, pomacentrids larvae showed no significant cardinal orientation in ocean water (Fig. 4A, Table 1).

## Discussion

The concept of ‘taking the lab to the ocean’ was realized by mounting a behavioral chamber and an imaging system on a drifter equipped with an environmental sensing system. This novel system allowed us to observe differences in larval swimming speed and orientation inside and outside reef water plumes. These distinct motions could be caused by differences in chemical signature of the water, rather than temperature or current direction. Odor choice tests established that larval reef fishes can recognize reef odor and prefer it over ocean water [15] but could not have made such a connection between odor detection and swimming behavior. The odor choice studies were done with larvae from the same two fish families, cardinalfish (Apogonidae) and damselfish (Pomacentridae), and with water from the same location, but only in the current study could the larvae express differences in orientation responses. This represents the first evidence of an *in situ* behavioral response to reef odor by coral reef fish larvae and supports the idea that a non-directional stimulus (odor) can trigger a directional response.

It is important to note that both odor choice studies and ours concluded that temperature, normally a strong signal in the ocean, did not appear to influence the observed behavior (Fig. 5). Thus odor rather than temperature caused swim speed and orientation to be different in plume and ocean water. In addition, wind and current direction did not correlate with orientation direction (Fig. 4C–D). This leaves the presence of a reef crest as the only tested feature correlated with swimming orientation (Fig. 4B). Previous work following damselfish larvae of the black axil chromis (*Chromis atripectoralis*) by observers equipped with scuba found a South-Southeast larval orientation on the leeward and windward sides of Lizard Island during the day [35]. Such location-independent orientation excludes the use of a directional cue such as reef sound. Here damselfish larvae swam alongshore from both northern and southern shore of One Tree Island reef, which indicated that they had a strong sense of direction of the reef. The reef crest is a natural source of sound [36], [37] and our test deployment distance from the reef crest falls within the expected range for orientation to directional sound, estimated a 1 km distance limit for directional hearing in larval fishes [20]. Various studies have implicated sound as an orientation cue to fish larvae for reef location [13], [18], [19]. The present work suggests that damselfish larvae detected the location of the reef, possibly through sound, within *ca*. 1 km from the reef crest (Fig. 2). Tackling the distance perception of reef ambient sound levels may thus require *in-situ* orientation experiments in absence of noise (e.g., scuba bubbles), in conjunction with propagation experiments of the reef soundscape [20], [37].

Most fish larvae recognized the odor plume, yet their response varied among families: contrary to apogonids, more pomacentrid larvae were directional when in the plume, yet less active (Table 1, Fig. 3, SI1). The ultimate cause and behavioral-ecological interpretation of these responses will require further study. We can speculate that the damselfish response to swim slower is a primitive kinesis behavior that is helpful in staying within the odor cue. Swimming westward indicates a switch to a different cue (i.e., the larva inside the DISC’s arena is drifting together with the water mass thus cannot experience a spatial gradient); this cardinal orientation would prevent being carried out into the open ocean to the East (Fig. 2), which seems to be an advantageous strategy. One would then like to know why cardinalfish do not show this response. The swim speed of cardinalfish increased in the odor plume without showing any specific orientation (Fig. 3, SI1). Indeed, different responses to odor detection are possible [38] and cardinalfish increased activity may reflect infotaxis behavior, which integrates spatial odor information [39]. They may have tried to find spatial differences in odor patches that exist outside the behavioral chamber, but the enclose prevent them to orient accordingly. Cardinalfish larvae could use such searching strategy to integrate sparse odor information and/or map the reef-born odor plume. However, because fish larvae are too small to be tagged by conventional means, this theory can only be tested in the laboratory [39]. The swim speed of cardinal fish increased significantly in the odor plume (Fig. 3, SI1), although lower than their critical speed measured in the laboratory (*ca*. 20 cm.s^−1^) [29]. In the DISC they swam an order of magnitude slower (Fig. 3). It is obvious that larvae cannot maintain a sustained swim speed in a relatively small behavioral arena of 38 cm in diameter, yet larval fish modified their activity with burst speeds of *ca*. 10 cm.s^−1^ across the arena (Fig. S1), which resulted in significant differences in swimming speed between plume water compared to ocean water (Table 1). Both observed responses to odor by damselfish and cardinalfish could be adaptive to maintain a position close to the reef.

It remains important to point out that spatial odor gradients at spatial scales of reefs are unlikely to be informative for orientation of cm-scale fish larvae with mm-scale nares separation: larval fishes and even large fishes cannot detect spatial odor concentration gradients that could direct them toward the reef. However, temporal information related to swimming patterns and turning rates might provide small-scale directional information via infotaxis [39], even with mm-scale nares separation. Sharks use bilateral odor arrival time differences [40], with which they steer into a patch of food odor, thereby improving their chance to remain connected to the large scale food odor plume. While nares separation in fish larvae is much smaller than in sharks, even millisecond arrival time differences can be processed by animal brains [38]. As the larvae recognize the odor of the home reef [16] this simple patch orientation mechanism might help them to stay within the reef odor halo. The smell of reefs seems to play a role in a larval navigational system, allowing pelagic larvae to find coral reefs in general, possibly natal reefs.

By using the term “navigation”, we assume that larval fish are migrating back home (or to a place like home) from the open ocean. This requires both a sense of direction (compass) and geographic location (map) or/and access to familiar cues. Discrete reef odor sources could be determined by associating particular odors with the ebb flow coming from different locations of One Tree Island, or from other islands of the Capricorn Bunker Reef [16]. Thus, olfactory cues may not be limited to the extent of the tidal influence and associated eddy field (a few tens of kilometers offshore), but could extend to spatial scales corresponding to a network of reefs’ tidal halos (up to a few hundreds of kilometers). Such odor signal can be extrapolated to unfamiliar areas, not only home. Alternatively, home-recognition cues, presumably imprinted at or shortly after birth in demersal spawners (e.g., damselfish) or mouth brooders (e.g., cardinalfish), may be independent of map cues used to derive geographic position relative to the settlement habitat. Further manipulation of home-recognition cues may be necessary to distinguish between the two.

Our new information would add reef odor to larval transport and recruitment models [41], [14] as a non-directional signal of reef proximity, detectable everywhere in the reef odor halo generated by the oscillating tides [42]. It supports the possibility that larvae retained in the halo can then use directional sound to determine the reef location for final entry into the reef structure. Additional experiments are necessary to test this hypothesis. Our novel approach demonstrates that recording larval fish movement in their natural setting gives unique information on behavioral decisions, and underlines the critical importance of *in situ* Lagrangian observations.

In sum, we propose that (home) reef odor acts as a wake up call, or a signal informing the animal to modify its swimming speed and to switch to other sensory information for direction toward the reef. Both types of responses to reef-born odor plume, i.e., reducing swimming speed and sensory switching by orienting alongshore or mapping olfactory signal by infotaxis, would increase the chances of successful settlement. We can only speculate about the nature of other sensory signals (e.g., sound, polarized light, celestial cues, magnetic field, wave and wind patterns) as the sequence of settlement cues needs further observations. Cue manipulation *in situ* will be necessary to elucidate the sensory processing sequence of pre-settlement fish larvae; for example, introducing reef odor into the behavioral arena set adrift in blue waters, outside the tidal halo, will help isolate the larva’s response from that to reef sounds. Importantly, the central role of chemo-sensory information and odor maps in the early life history of fish raises concerns of compounded effects of habitat fragmentation, ocean acidification and chemical pollution altering olfactory discrimination [43] and disturbing the signaling environment [44].

## Supporting Information

Figure S1 **Movement analysis of individual fish larvae deployed in the Drifting In Situ Chamber (DISC) at One Tree Island (OTI) on the Great Barrier Reef, February of 2009.** A) cardinalfish larva of the species *Cheilodipterus quinquelineatus* (Family: Apogonidae) in plume water (deployment #55); B) cardinalfish larva *C. quinquelineatus* in ocean water (deployment #38); C) damselfish larva of the species *Pomancentrus moluccensis* (Family: Pomacentridae) in plume water (deployment #1); D) damselfish larva P. coelestus in ocean water (deployment #16). Subplots represent: a) density distribution of the fish larva’s swimming speeds; b) fish larva’s original trajectory in the chamber’s frame of reference; c) fish larva’s trajectory in the cardinal reference, i.e., corrected by the compass rotation. The larva’s movement is sampled every second for a total of 15 minutes (or 900 s); the larva’s trajectory is color-coded by time in seconds. The x-axes on subplots (a) have different scales since damselfish are faster swimmers than cardinalfish.
(PDF)

Text S1 **Statistical Analyses.**
(DOC)
