# Supplementary figures and images for: Reef Odor: A Wake Up Call for Navigation in Reef Fish Larvae
Source: PLoS One. 2013 Aug 28;8(8):e72808. doi: 10.1371/journal.pone.0072808 (PMC3755995; doi:10.1371/journal.pone.0072808)

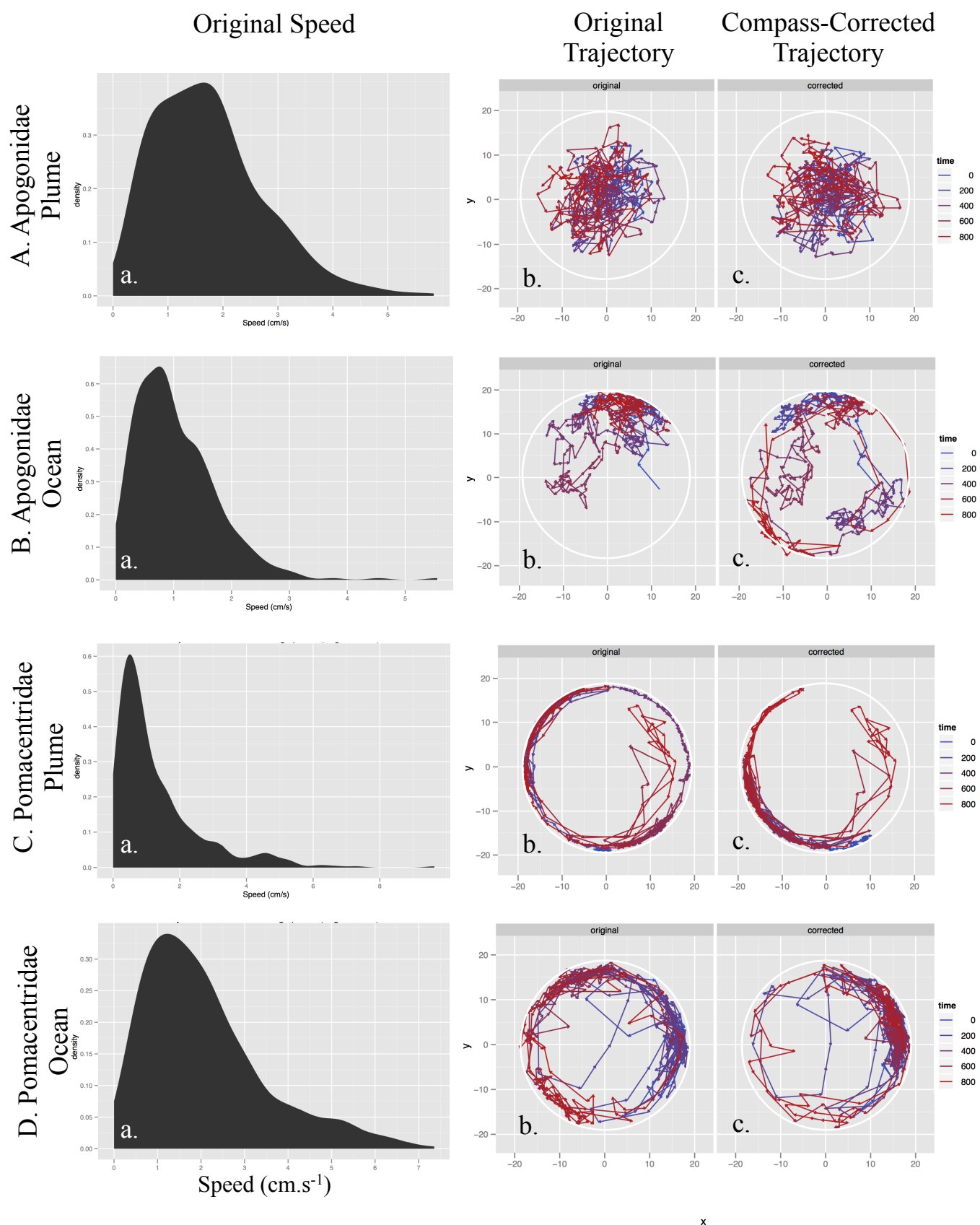

**Figure S1**

Supplement: Figure S1 — Movement analysis of individual fish larvae deployed in the Drifting In Situ Chamber (DISC) at One Tree Island (OTI) on the Great Barrier Reef, February of 2009. A) cardinalfish larva of the species Cheilodipterus quinquelineatus (Family: Apogonidae) in plume water (deployment #55); B) cardinalfish larva C. quinquelineatus in ocean water (deployment #38); C) damselfish larva of the species Pomancentrus moluccensis (Family: Pomacentridae) in plume water (deployment #1); D) damselfish larva P. coelestus in ocean water (deployment #16). Subplots represent: a) density distribution of the fish larva’s swimming speeds; b) fish larva’s original trajectory in the chamber’s frame of reference; c) fish larva’s trajectory in the cardinal reference, i.e., corrected by the compass rotation. The larva’s movement is sampled every second for a total of 15 minutes (or 900 s); the larva’s trajectory is color-coded by time in seconds. The x-axes on subplots (a) have different scales since damselfish are faster swimmers than cardinalfish. (PDF) [file pone.0072808.s001.pdf]
