# Supplementary material for: Reef Odor: A Wake Up Call for Navigation in Reef Fish Larvae
Source: PLoS One. 2013 Aug 28;8(8):e72808. doi: 10.1371/journal.pone.0072808 (PMC3755995; doi:10.1371/journal.pone.0072808)
Supplement: Text S1 — Statistical Analyses. (DOC) [file pone.0072808.s002.doc]

TEXT S1

Statistical Analyses

1. Extraction of data from each larva - Circular statistics treat data as independent unit vectors pointing toward recorded angles (Batschelet 1981). The first level of the analysis is to assess the distribution of angles for each larva. Each sum vector is tested for significant directionality (i.e. concentration of angles around one direction) with Rayleigh’s test. However, while successive swimming directions are independent (lags ≥ 1 s shows auto-correlation < 10%), positions are not. Indeed, when observed at one or two seconds intervals, the larva is likely to remain in the same region of the arena. Therefore, positions are subsampled every 10 s which, given their average swimming speed in the arena, is enough for them to move across its diameter, and Rayleigh’s test is performed on this subsample of independent data. After computing the statistics on the subsample taken at 1, 11, 21, etc. seconds, they are recomputed on the subsample shifted to 2, 12, 22, etc. seconds, and so on until 10, 20, 30, etc. The final statistics (mean angle, variance, Rayleigh’s R, and p-value of the test) are computed as means of the statistics computed these ten independent subsamples of data. Larvae show significant directionality when p ≤ 0.05.

2. *Analysis of population level trends* - The second level of the analysis is to test whether larvae orient consistently as a population. The data now consists of the mean angles of orientation of larvae which were directional and were not found to display artifactual or routine swimming. Such angles are, once again, tested for directionality with Rayleigh’s test. Significant directionality at the population level denotes orientation in relation to a common cue. To test what environmental factors influence orientation, directionality statistics can be compared between conditions (sunny/cloudy days, single individual/groups of larvae, locations around a reef etc.).

First, the precision of orientation, represented by the Rayleigh’s number (R), is compared with a Wilcoxon-Mann-Whitney test to compare two conditions (i.e., ocean and plume water) which is not sensitive to the fact that R is bounded between 0 and 1. Second, the actual angle of orientation can be used to test for the influence of a clue of known direction (e.g. current and wind direction, direction the island). The Rayleigh’s test can be used as a test of the conformance of observed orientation angles to an angle determined *a priori*. We tested whether larvae swam in a direction with respect to the island and the direction of the wind, subtracting the angle of the target to the angle of orientation and to test the conformance of the difference to zero. We also regressed the angle of orientation on the angle of the supposed cue using a circular regression.

Swimming speed (a measure of the activity of the larva in the chamber) was compared in different conditions. A *t*-test/Anova was used to compare swimming speeds in and out of a plume of odor emanating from the reef.

All analyses are performed using R and in particular the *circular* package (Agostinelli 2007-08-15).

References

C. Agostinelli. Package circular 0.3-8: Circular statistics. Online reference, 2007-08-15. URL <http://cran.r-project.org/web/packages/circular/index.html>.

E. Batschelet. *Circular statistics in biology*. Academic Press, London, 1981.
